# Restructuring the Obesity Paradigm: Molecular Etiologies, Clinical Complexities, and the Future of Precision Intervention

**DOI:** 10.7759/cureus.106767

**Published:** 2026-04-10

**Authors:** Tint S Latt, Than T Aye, Ko Ko, San S Win, Tet T Chit, Kyar Nyo S Myint

**Affiliations:** 1 Department of Diabetes and Endocrinology, University of Medicine 2, Yangon, MMR; 2 Department of Diabetes and Endocrinology, Grand Hantha International Hospital, Yangon, MMR; 3 Department of Medicine, University of Medicine 2, Yangon, MMR; 4 Department of Endocrinology, University of Medicine 2, Yangon, MMR; 5 Department of Diabetes and Endocrinology, University of Medicine Mandalay, Mandalay, MMR; 6 Third Department of Medicine, Yangon General Hospital, Yangon, MMR

**Keywords:** gut microbiome, lifestyle intervention, obesity, pharmacotherapy, precision medicine, weight stigma

## Abstract

Obesity is a complex, chronic, and relapsing disease affecting adults globally and continues to rise across all age and socioeconomic groups. Once regarded as a lifestyle issue, obesity is now recognized as a multifactorial condition influenced by multiple factors. This review consolidates current evidence on the dynamic pathophysiology of obesity, highlighting advances in understanding its genetic foundations, hormonal imbalances, gut microbiome disruptions, and the influence of early-life exposures, and emphasizing the systemic impact of obesity through its associations with cardiometabolic diseases, certain cancers, and mental health disorders. Emerging treatment paradigms include glucagon-like peptide-1 and dual agonists, endoscopic and surgical interventions, and microbiota-directed therapies. Advances in artificial intelligence and precision medicine are also redefining individualized care. Despite these developments, obesity remains underdiagnosed and undertreated in many healthcare systems. In high-income regions, the uptake of advanced therapies is limited by fragmented care pathways, in which obesity management is spread across multiple uncoordinated providers, and by affordability barriers due to high costs. In low-resource settings, constrained infrastructure and competing health priorities continue to hinder timely diagnosis and treatment. A shift toward holistic, patient-centered, and equity-driven models of care is essential for effectively addressing the global burden of obesity and improving long-term health outcomes.

## Introduction and background

Obesity has emerged as a critical public health issue in the 21st century, with the World Health Organization (WHO) reporting that over 2.5 billion adults are overweight and approximately 890 million individuals meet criteria for obesity. It is now recognized as a serious, chronic, progressive, relapsing, and treatable multifactorial neurobehavioral disease, wherein an increase in adiposity promotes adipose tissue dysfunction and abnormal fat-mass-related physical forces, resulting in adverse metabolic, biomechanical, and psychosocial health consequences [1]. The global prevalence has more than doubled since 1990 and nearly tripled since 1975, indicating a dramatic rise in obesity-related morbidity and mortality worldwide [2]. The burden of obesity is unevenly distributed, with low- and middle-income countries (LMICs) increasingly affected by the dual challenge of undernutrition and rising obesity prevalence. In Southeast Asia and Latin America, rapid urbanization, dietary shifts, and declining physical activity have accelerated this trend [3,4]. In contrast, resource-rich settings face a parallel challenge due to excess availability and consumption of ultraprocessed foods (UPFs), sedentary lifestyles, and obesogenic environments, highlighting that the epidemic spans both extremes of the economic spectrum. Equally concerning is the rising incidence of pediatric obesity, which is associated with early-onset cardiometabolic disorders and high risk of adult obesity persistence [5]. Leading organizations, including the American Medical Association and World Obesity Federation, have formally endorsed this disease classification [6-8]. It also mandates a more systematic approach to diagnosis, management, and long-term follow-up. Moreover, classifying obesity as a disease strengthens the rationale for allocating healthcare resources and addressing the persistent stigma faced by individuals with obesity, both clinically and socially [9].

Despite rising prevalence, obesity remains underdiagnosed and undertreated in many health systems. Most individuals meeting diagnostic thresholds as per the International Classification of Diseases, 10th Revision, are neither identified nor do they receive appropriate intervention [10,11]. This treatment gap reflects inadequate clinical training, systemic weight bias, and resource constraints. Reframing obesity as a chronic disease is central to fostering proactive screening, individualized care pathways, and policy-level responses, including insurance coverage, research prioritization, and structural interventions [12].

Historically, obesity has been managed under the energy balance model, which posits that weight regulation is governed by caloric intake and expenditure. While this framework remains conceptually relevant, it oversimplifies a highly regulated, biologically defended process [13]. Evidence now implicates mechanisms such as adaptive thermogenesis, neuroendocrine feedback (e.g., leptin and ghrelin), hypothalamic plasticity, and host-microbiota interactions in establishing a higher defended body weight set point, contributing to weight regain and therapeutic resistance [14,15].

Body mass index (BMI), though widely used, is a limited metric that fails to distinguish lean mass from adiposity, does not reflect fat distribution, and correlates poorly with metabolic risk in certain populations [16]. Notably, individuals with normal BMI may exhibit metabolic dysfunction, while some individuals with elevated BMI may remain metabolically healthy (metabolically healthy obesity) [17]. More nuanced phenotyping, incorporating waist circumference, body composition, visceral adiposity, insulin resistance indices, and inflammatory biomarkers, offers improved risk stratification. These measures are integral to the adiposity-based chronic disease model, which incorporates metabolic burden and functional impairment into clinical decision-making [18,19]. Traditional treatment approaches, progressing from lifestyle changes to drugs and then surgery, may delay effective care by overlooking individual variability in disease severity, psychological readiness, and biological drivers [19]. In contrast, patient-centered models promote early risk stratification and personalized interventions [20]. Recent studies have identified discrete obesity subtypes based on neuroendocrine, behavioral, and genetic profiles, suggesting the potential for targeted interventions tailored to distinct pathogenic mechanisms [21,22]. In parallel, artificial intelligence and machine learning methodologies are increasingly being leveraged to optimize care pathways and predict therapeutic response using real-world data analytics [23].

**Figure 1:**
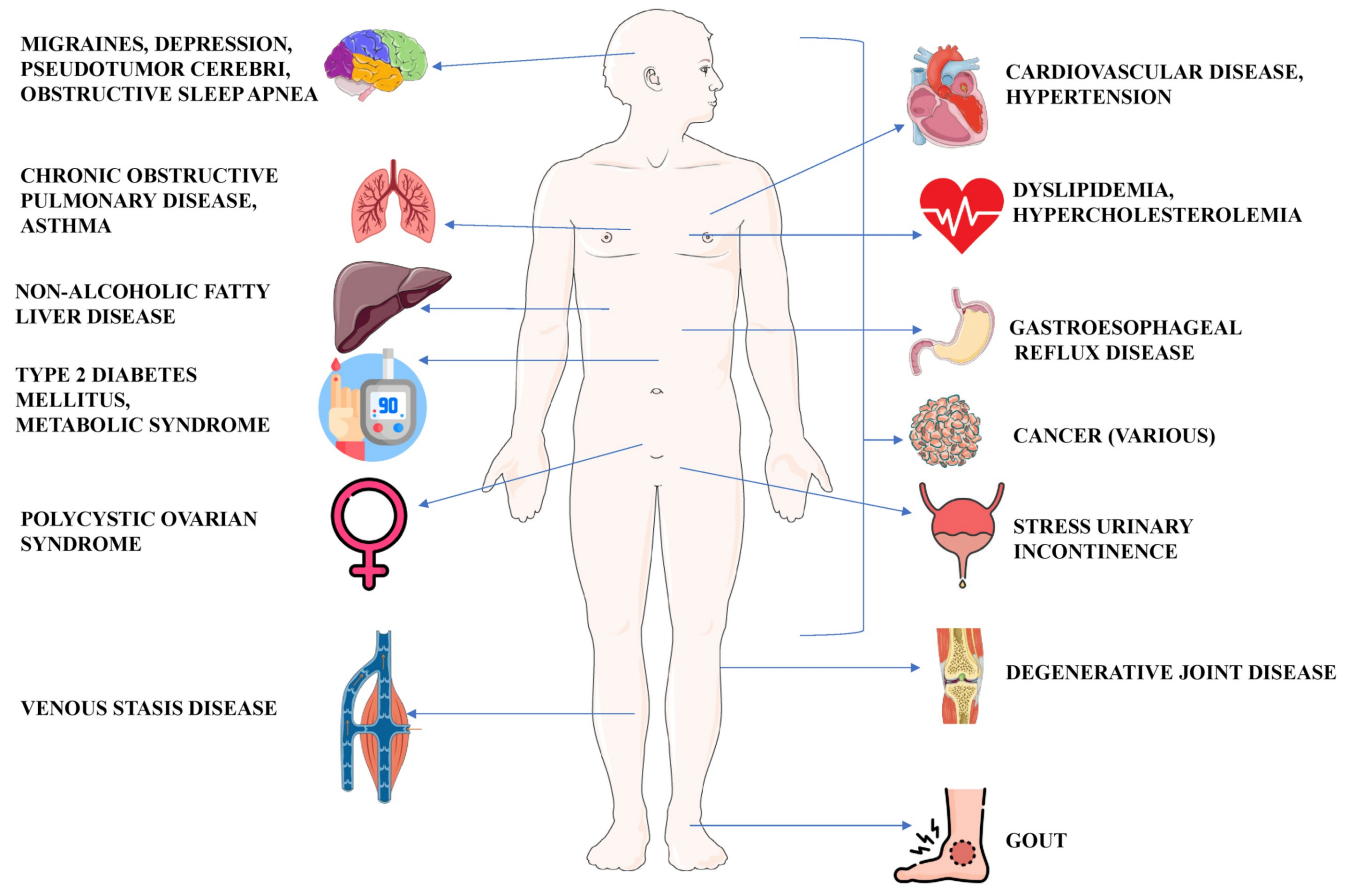
The obesity comorbidity web Image credit: This is an original image created by the author Tint S. Latt using Microsoft PowerPoint (Microsoft Corporation, Redmond, WA)

This article is a narrative review to provide a synthesis of evidence from basic, translational, and clinical domains to elucidate the multifactorial pathogenesis of obesity and the evolution of its therapeutic landscape. It examines genetic and hormonal mechanisms (e.g., leptin, ghrelin, insulin, and gut-brain axis), environmental determinants (diet, physical inactivity, sleep, and socioeconomic status), and obesity-related comorbidities, including cardiometabolic disease, metabolic dysfunction-associated steatotic liver disease (MASLD), malignancies, and mental health conditions (Figure 1). Current and emerging interventions, ranging from behavioral and pharmacologic treatments to metabolic surgery, are discussed in the context of precision medicine, with an emphasis on phenotypic subtyping, microbiome-targeted strategies, and artificial intelligence.

## Review

Evolving pathophysiology of obesity

Obesity is increasingly recognized as a complex, multifactorial disease influenced by genetic, neuroendocrine, and microbial determinants. Genetic architecture accounts for 40%-70% of BMI variability, with monogenic mutations in the leptin-melanocortin pathway (e.g., melanocortin-4 receptor), syndromic conditions such as Prader-Willi, and a broad polygenic background (e.g., FTO, >500 genome-wide association study loci) shaping susceptibility across populations [24]. Hormonal drivers, including leptin, ghrelin, glucagon-like peptide-1 (GLP-1), insulin, cortisol, and sex hormones, orchestrate appetite regulation and energy balance; disruptions lead to leptin and insulin resistance, rebound hyperphagia, adverse fat distribution, and metabolic dysfunction [25]. The gut microbiome further contributes through dysbiosis, energy harvest, low-grade inflammation, and altered gut-brain signaling, amplifying obesity risk [26]. Collectively, these interconnected pathways highlight the biological resistance to weight loss and underscore the need for therapies that target multiple axes simultaneously (Table 1).

**Table 1:** Integrated drivers of obesity pathophysiology LEP: leptin gene; LEPR: leptin receptor; MC4R: melanocortin-4 receptor; FTO: fat mass and obesity associated; TMEM18: transmembrane protein 18; BMI: body mass index; GLP-1: glucagon-like peptide-1; NPY: neuropeptide Y; AgRP: agouti-related peptide; LPS: lipopolysaccharide; SCFA: short-chain fatty acid

Category	Key features/roles	Examples/sources	Alterations in obesity
Genetics	Monogenic (rare), syndromic, polygenic inheritance	LEP, LEPR, MC4R, FTO, TMEM18	Monogenic rare (<5%); polygenic common; ethnic variability in BMI cutoffs
Hormones	Appetite, satiety, and metabolism regulation	Leptin, Ghrelin, GLP-1, insulin, cortisol, sex hormones	Leptin and insulin resistance, rebound ghrelin rise, cortisol excess, altered fat distribution
Neuroendocrine	Hypothalamic regulation of energy balance	POMC/CART, NPY/AgRP circuits	Impaired central satiety signaling, increased orexigenic drive
Gut microbiome	Energy harvest, SCFA production, gut-brain axis	Firmicutes/bacteroidetes ratio, SCFAs, LPS endotoxemia	Enhanced lipogenesis, chronic inflammation, and altered satiety signaling

Environmental and behavioral contributors

Obesity is shaped by environmental and behavioral determinants that act alongside biological predisposition. Diets dominated by UPFs and high in sugars, fats, and additives promote passive overconsumption through hyperpalatability, impaired satiety, and metabolic disruption, with robust evidence linking UPF intake to weight gain and obesity [27]. Sleep deprivation and circadian misalignment further dysregulate leptin, ghrelin, cortisol, and glucose metabolism, predisposing to increased appetite, hedonic food choices, and insulin resistance [28]. Modern sedentary lifestyles compound these risks: prolonged screen time fosters inactivity and exposure to obesogenic marketing, while hedonic eating driven by mesolimbic reward pathways perpetuates cravings for calorie-dense foods. Early-life exposures also play a decisive role: maternal obesity, gestational diabetes, birthweight extremes, rapid catch-up growth, and formula feeding increase later obesity risk; while breastfeeding and responsive caregiving confer modest protection [29]. Collectively, these influences underscore the interplay between food systems, lifestyle patterns, neurobehavioral drivers, and early-life programming, reinforcing the need for preventive strategies spanning policy, healthcare, and community interventions (Table 2).

**Table 2:** Environmental and behavioral drivers of obesity RCT: randomized controlled trial; DOHaD: Developmental Origins of Health and Disease

Category	Key features/mechanisms	Evidence/examples	Impact on obesity	References
Ultraprocessed foods	High in refined carbs, fats, sugars, additives; hyperpalatable	RCTs and cohort studies show ↑ caloric intake and weight gain	Excess energy intake, impaired satiety, altered microbiota	[27]
Sleep and circadian rhythm	Short sleep, irregular schedules, hormonal dysregulation	Meta-analyses: 30%–55% ↑ obesity risk	↑ Ghrelin, ↓ Leptin, cortisol disruption, insulin resistance	[28]
Sedentary lifestyle and hedonic eating	Low energy expenditure, high screen time, reward-driven eating	Observational and neuroimaging studies	Positive energy balance, food cravings, habitual overeating	[29]
Early-life exposures	Maternal obesity/diabetes, birthweight extremes, feeding practices	DOHaD framework; first 2,000 days critical	Long-term metabolic programming, ↑ childhood/adult obesity	[29]

Psychosocial, intergenerational, and societal impacts of obesity

Obesity exerts profound effects that extend beyond physical health into mental well-being, social participation, family health, and economic stability. Individuals with obesity face a disproportionately high burden of psychiatric comorbidities, including depression, anxiety, binge eating disorder, and attention deficit/hyperactivity disorder, which often act bidirectionally with obesity through emotional eating, medication effects, and stigma [30]. Quality of life is consistently reduced, with impairments in physical, emotional, and social domains, particularly among children and adolescents who experience bullying and peer victimization. Weight bias further compounds this burden, as discrimination is pervasive in healthcare, education, and employment, leading to delayed care, social exclusion, and maladaptive coping behaviors that reinforce weight gain. The intergenerational impact of obesity is equally significant, with maternal and paternal obesity programming future metabolic risk in offspring via intrauterine exposures and epigenetic mechanisms. Shared household environments perpetuate cycles of obesity across generations [31]. At the societal level, obesity imposes staggering economic costs, exceeding $2 trillion globally each year, driven by direct healthcare expenditures and indirect losses from absenteeism, reduced productivity, disability, and premature mortality [32]. Relying on BMI as a universal health metric leads to missed diagnoses and overlooked iatrogenic harm [33]. Addressing these psychosocial, intergenerational, and economic dimensions requires comprehensive solutions that prioritize stigma reduction, equitable access to care, family-centered prevention, and structural policy reforms (Table 3).

**Table 3:** Barriers to effective obesity care and proposed solutions BMI: body mass index

Barrier	Description	Academic research to address the barriers	Reference
Weight stigma	Bias in healthcare and society	Evaluating the impact of provider education and person-first communication on patient engagement and outcomes	[30]
Limited access	High cost, poor infrastructure, limited insurance	Research assessing cost-effectiveness and access models	[31]
Treatment attrition	Dropouts from lifestyle or drug programs	Care models to improve long-term adherence	[32]
Overreliance on BMI	BMI not reflective of metabolic risk	Body composition metrics and metabolic biomarkers as complementary risk stratification tools	[33]

Treatment paradigms: current strategies and limitations

Lifestyle Modifications and Behavioral Therapy

Lifestyle intervention remains the cornerstone of obesity treatment, and when implemented effectively, it can lead to clinically meaningful weight loss and improvement in metabolic health. Core components include dietary modification, increased physical activity, sleep hygiene, stress reduction, and behavioral change strategies [34].

Dietary interventions range from calorie-restricted and low-fat diets to low-carbohydrate, Mediterranean, and intermittent fasting approaches. Meta-analyses suggest that no single diet is superior across all populations; instead, long-term adherence, caloric deficit, and nutritional adequacy are the most consistent predictors of success (Table 4) [35].

**Table 4:** Comparison of major dietary interventions in obesity

Diet type	Core features	Evidence for weight loss	Key benefits/challenges	Reference
Mediterranean	High in fruits, vegetables, and olive oil	Sustained	Cardioprotective	[36]
Low carbohydrate	Reduced carb intake, higher fat/protein	Short to medium term	Adherence issues	[37]
Intermittent fasting	Time-restricted feeding	Emerging evidence	Hunger, compliance	[38]

Physical activity recommendations typically emphasize 150-300 minutes per week of moderate-intensity exercise, combined with resistance training to preserve lean body mass. However, the compensatory increase in appetite and energy intake in response to exercise may limit weight loss unless supported by concurrent dietary strategies [39].

**Figure 2:**
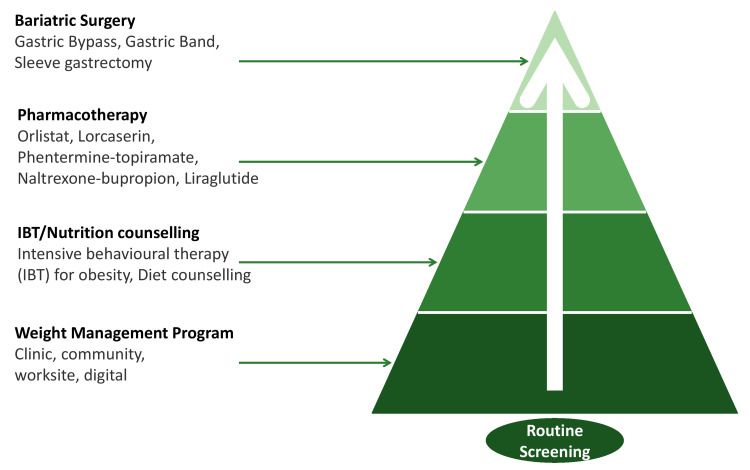
Management framework for obesity, highlighting the progressive approach from weight management programs and nutrition counseling to pharmacotherapy and bariatric surgery, built upon the foundation of routine screening IBT: intensive behavioral therapy Image credit: This is an original image created by the author Tint S. Latt using Microsoft PowerPoint (Microsoft Corporation, Redmond, WA)

Behavioral therapy forms the backbone of sustainable weight management. Techniques such as self-monitoring, goal setting, problem-solving, stimulus control, and cognitive restructuring are delivered through individual or group counseling, often led by dietitians, psychologists, or trained coaches. Digital health tools, including mobile apps, wearable trackers, and virtual coaching platforms, have expanded access to behavioral support and may enhance engagement and adherence [40]. Despite their efficacy, lifestyle interventions are challenged by high attrition rates, weight regain, and limited accessibility in many healthcare settings (Figure 2).

Pharmacotherapy

**Figure 3:**
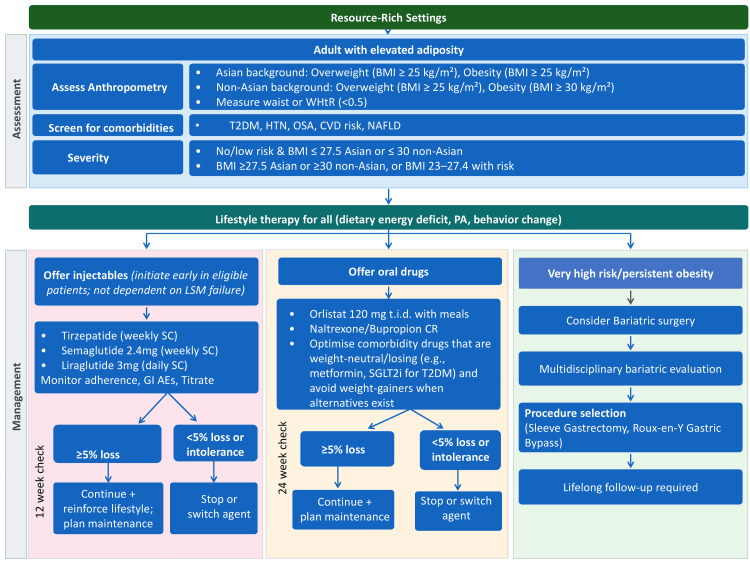
Flowchart of obesity management as per guidelines for resource-rich settings BMI: body mass index; SGLT2i: sodium-glucose cotransporter 2 inhibitors; WHtR: waist-to-height ratio; T2DM: type 2 diabetes mellitus; HTN: hypertension; OSA: obstructive sleep apnea; CVD: cardiovascular disease; NAFLD: nonalcoholic fatty liver disease; PA: physical activity; LSM: lifestyle management; GI: gastrointestinal; AEs: adverse events Image credit: This is an original image created by the author Tint S. Latt using Microsoft PowerPoint (Microsoft Corporation, Redmond, WA) based on the data from [38,43,44]

**Figure 4:**
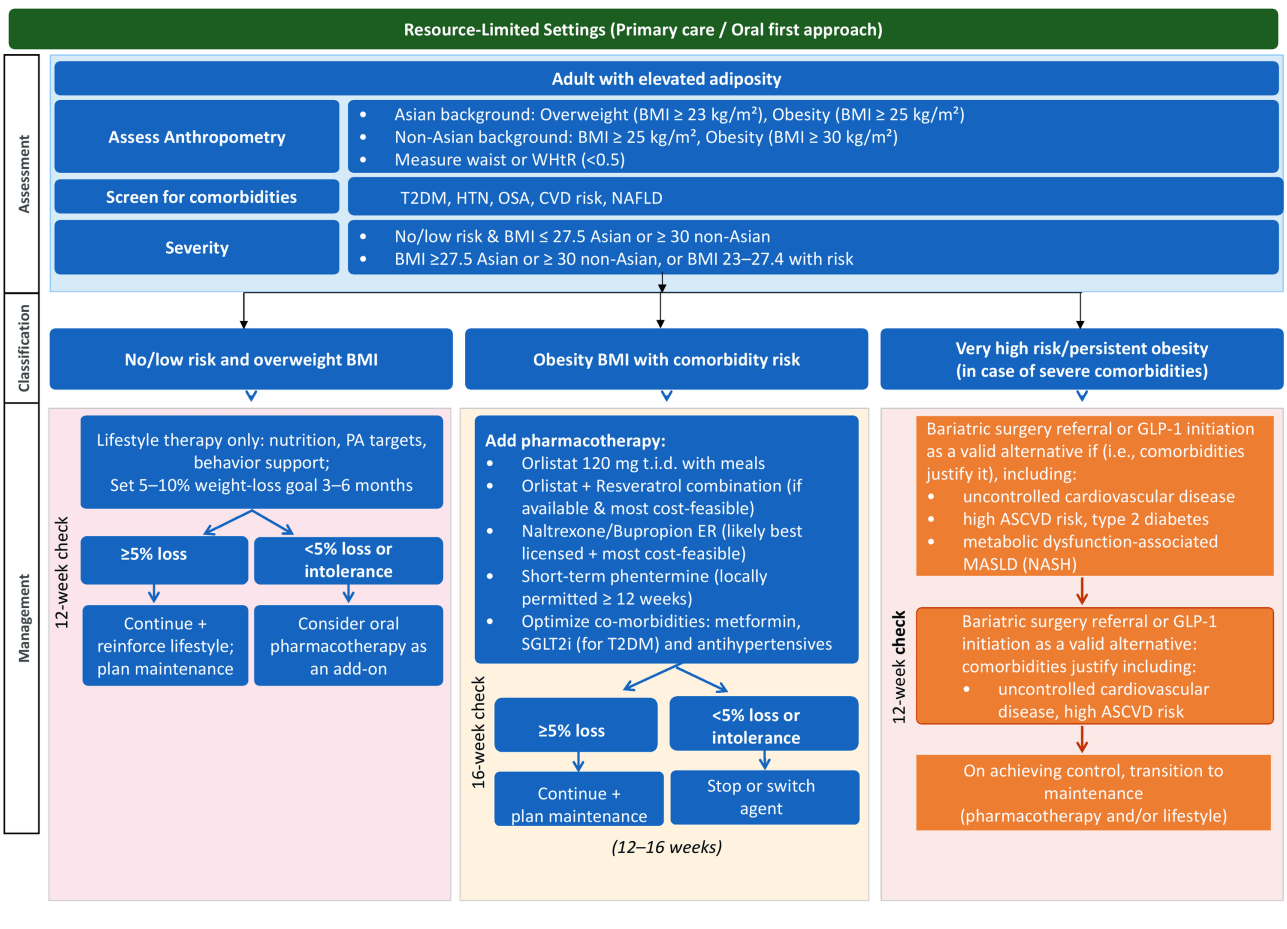
Flowchart of obesity management as per guidelines for resource-limited settings T2DM: type 2 diabetes mellitus; HTN: hypertension; OSA: obstructive sleep apnea; CVD: cardiovascular disease; NAFLD: nonalcoholic fatty liver disease; WHtR: waist-to-height ratio; BMI: body mass index; SGLT2i: sodium-glucose cotransporter 2 inhibitors; GLP-1: glucagon-like peptide-1; ASCVD: atherosclerotic cardiovascular disease; MASLD: metabolic dysfunction-associated steatotic liver disease; NASH: nonalcoholic steatohepatitis Image credit: This is an original image created by the author Tint S. Latt using Microsoft PowerPoint (Microsoft Corporation, Redmond, WA)

Pharmacologic therapy is recommended for individuals with obesity who fail to achieve or sustain clinically meaningful weight loss of ≥5% through lifestyle modification (according to the International Diabetes Federation (IDF) Global Clinical Practice Recommendations for Diabetes (2025)) alone and who meet eligibility criteria based on BMI and comorbidity status (Figures 3, 4). Pharmacotherapy serves as an adjunct to, not a replacement for, behavioral interventions, and its goal is not merely weight loss but improvement in cardiometabolic health and quality of life (Table 5) [41,42].

**Table 5:** Antiobesity injectable medications GLP-1: glucagon-like peptide-1; GIP: glucose-dependent insulinotropic polypeptide

Medication	Mechanism of action	Dosing	Reported weight loss	Key adverse events	References
Liraglutide	GLP-1 receptor agonist; reduces appetite, delays gastric emptying	3.0 mg daily (subcutaneous)	8%-10% of baseline body weight	Acute kidney injury. gastrointestinal adverse effects like nausea, vomiting, hypoglycemia	[43]
Semaglutide	GLP-1 receptor agonist; reduces appetite, delays gastric emptying	2.4 mg weekly (subcutaneous)	10%-15% of baseline body weight	Gall bladder diseases, acute kidney injury, thyroid C-cell tumor risk, gastrointestinal adverse effects	[43]
Tirzepatide	Dual GLP-1 and GIP receptor agonist; enhances satiety and insulin sensitivity	Weekly injection 15 mg (subcutaneous)	Up to 22% of baseline body weight	Gastrointestinal intolerance, pancreatitis, hypoglycemia	[44,45]
Emerging polyagonists	GLP-1/GIP/glucagon or GLP-1 + amylin analogues; multipathway targeting	Under investigation	>15%-24% (in early trials)	Gastrointestinal intolerance, reduced appetite, gall bladder disease, mild increase in heart rate	[46]

Figure 3 outlines guideline-based obesity management in resource-rich settings (National Institute for Health and Care Excellence (NICE) 2025, IDF 2025, American College of Cardiology 2025) [47-49]. Assessment includes ethnic-specific BMI thresholds and comorbidity profiling, recognizing elevated risk at lower BMI in Asian populations. Lifestyle modification remains the foundation, but newer consensus supports early initiation of GLP-1/glucose-dependent insulinotropic polypeptide (GIP) receptor agonists without requiring prior lifestyle failure. Agents such as tirzepatide, semaglutide, and liraglutide are preferred in specialist services, with continuation based on a six-month response. Oral therapies (e.g., orlistat, naltrexone/bupropion) remain alternatives, with comorbidity drugs optimized to avoid weight gain. In very high-risk or persistent obesity, multidisciplinary bariatric surgery referral is appropriate, though GLP-1 initiation is also recommended for patients with cardiovascular disease or metabolic dysfunction-associated steatohepatitis. Long-term management emphasizes maintenance through ongoing pharmacotherapy and lifestyle strategies, whereas Figure 4 [50-52] illustrates the management of obesity in resource-limited settings, aligned with Metabolic Syndrome and Excess Weight in East Asians Meeting 2011, IDF 2025, and NICE 2025 guidance. Lifestyle therapy remains the universal foundation, but oral pharmacotherapy is emphasized as a practical next step where resources are constrained. Orlistat remains the most accessible and cost-effective option, with continuation guided by the 12-week ≥5% weight-loss rule. In selected cases, combinations such as orlistat with resveratrol may be considered, alongside naltrexone/bupropion or short-term phentermine, where locally licensed. Optimizing comorbidity medications to avoid weight gain (e.g., metformin or sodium-glucose cotransporter 2 inhibitors in type 2 diabetes mellitus (T2DM)) is also critical. Bariatric surgery is recommended for very high-risk or persistent obesity, although access is often limited; in such situations, intensifying lifestyle support and community-based interventions become essential. This approach highlights a tiered, affordable strategy while retaining alignment with international guidelines.

Oral Pharmacotherapy in Resource-Limited Settings

In LMICs, the availability of injectable antiobesity medications is often constrained by cost, cold-chain requirements, and healthcare infrastructure. Oral pharmacotherapy remains an essential component of treatment in these settings and offers a more accessible, scalable solution.

Orlistat, the only FDA- and WHO-approved oral agent available without a prescription in some countries, inhibits gastrointestinal lipase and reduces dietary fat absorption by approximately 30%. While modest in its weight loss efficacy (~3%-5%), orlistat has demonstrated significant reductions in LDL cholesterol, blood pressure, and glycemic progression in patients with impaired glucose tolerance [53]. Gastrointestinal side effects, especially steatorrhea and fecal urgency, are common and can impair adherence. Though data are preliminary, these approaches exemplify regionally tailored solutions. Recent evidence from the multicenter Endocuff-assisted Colonoscopy Fecal Immunochemical Test randomized controlled trial (2025) demonstrated that combining orlistat with resveratrol resulted in significantly greater weight loss (-3.31 vs. -0.39 kg; p < 0.001), reductions in total body fat (-4.93%), diastolic blood pressure (-2.85 mmHg), and hepatic steatosis markers (controlled attenuated parameter score reduction of -23.5 dB/m; p < 0.05) compared with orlistat alone. The benefits were particularly notable in obese patients with steatosis, fibrosis, or type 2 diabetes. However, steatorrhea occurred in both groups, highlighting tolerability as an ongoing limitation [54].

Rational pharmacologic sequencing is critical. Oral agents may serve as first-line options in individuals with lower BMI or metabolic risk, with escalation to injectable or combination therapy guided by treatment response, tolerance, and patient preference. Efforts to improve access, including generic formulations, government subsidies, and inclusion in national essential medicine lists, can facilitate more equitable care (Table 6).

**Table 6:** Summary of oral antiobesity medications GI: gastrointestinal; TID: three times daily

Drug	Mechanism	Dosage	Average weight loss	Common side effects	Cost	Availability	References
Naltrexone-bupropion	Opioid/dopaminergic modulator	Titrated to 32/360 mg daily	~6%-8%	Nausea, insomnia, headache	Moderate	Limited	[53]
Orlistat + resveratrol	Combination: lipase inhibition (orlistat) plus antioxidant/anti-inflammatory modulation of adipogenesis (resveratrol)	Experimental/variable dosing in studies	~5%-7% (early clinical data)	GI upset (from orlistat), mild GI intolerance (resveratrol)	Low-moderate	Emerging, limited access	[54]
Orlistat	Lipase inhibitor	120 mg TID	~5%	GI distress, flatulence	Low	Widely available	[55]
Phentermine	Sympathomimetic appetite suppressant	15-37.5 mg/day	~5%-7%	Palpitations, insomnia	Low	Restricted (varies by country)	[56]

Bariatric and Endoscopic Interventions

For individuals with severe obesity (BMI ≥40 or ≥35 kg/m² with comorbidities) who have not achieved adequate results with conservative measures, bariatric surgery remains the most effective long-term treatment. Procedures such as Roux-en-Y gastric bypass and sleeve gastrectomy lead to sustained weight loss of 25%-35% and durable improvement or resolution of T2DM, hypertension, MASLD, and obstructive sleep apnea [57,58]. Beyond mechanical restriction and malabsorption, bariatric surgery exerts powerful hormonal effects. It enhances GLP-1 and peptide YY secretion, suppresses ghrelin, improves insulin sensitivity, and alters bile acid metabolism and gut microbiota, all contributing to its metabolic benefits independent of weight loss [59].

Endoscopic bariatric therapies (EBTs), such as intragastric balloons, endoscopic sleeve gastroplasty, and duodenal mucosal resurfacing, offer less invasive alternatives with fewer complications and shorter recovery times. Though their weight-loss efficacy is more modest (10%-15%), EBTs can serve as bridge therapies for high-risk patients or those unwilling to undergo surgery [60].

Despite their effectiveness, access to bariatric and endoscopic interventions is limited in many regions due to high upfront costs, lack of trained providers, and insurance barriers. Additionally, long-term outcomes are optimized when procedures are integrated into multidisciplinary care models involving nutritional counseling, psychological support, and close metabolic monitoring.

Future perspectives in obesity pharmacotherapy

GLP-1 receptor agonists remain the cornerstone, with novel high-dose oral formulations such as oral semaglutide 50 mg showing substantial weight loss and cardiometabolic benefits. Dual and triple agonists, including tirzepatide (GLP-1/GIP) and retatrutide (GLP-1/GIP/glucagon), are progressing through late-phase trials and indicate even greater efficacy [61]. The future of obesity management is being shaped by advances in pharmacotherapy, with emerging agents demonstrating efficacy that approaches bariatric surgery [62]. Combination therapies such as cagriSema (Novo Nordisk, Bagsvaerd, Denmark; GLP-1/amylin) further broaden the therapeutic potential, while oral nonpeptide GLP-1 receptor agonists like orforglipron and danuglipron offer improved convenience [63]. Beyond gut hormones, molecules such as bimagrumab and growth differentiation factor 15 analogs represent alternative mechanisms targeting body composition and appetite regulation [64]. Together, these therapies highlight a future where obesity treatment is increasingly personalized, effective, and accessible, with pharmacotherapy likely to play a central role in reducing the global burden of obesity (Table 7).

**Table 7:** Pipeline medications for obesity GLP-1: glucagon-like peptide-1; RA: receptor agonist; WL: weight loss; SC: subcutaneous; GIP: glucose-dependent insulinotropic polypeptide; IV: intravenous

Agent	Mechanism	Phase/status	Key findings	Route	References
Cagrilintide + semaglutide (cagriSema)	GLP-1 + amylin RA	Phase 3 ongoing	Enhanced satiety, durable WL	Injectable (SC)	[60]
Oral semaglutide 50 mg	GLP-1 RA (oral)	Phase 3 completed	~17% WL; cardiometabolic benefits	Oral	[61]
Orforglipron	Oral nonpeptide GLP-1 RA	Phase 2 positive	~15% WL; flexible dosing	Oral	[62]
Retatrutide	GLP-1 + GIP + glucagon RA	Phase 3 ongoing	Early data: >20% WL	Injectable (SC)	[62]
Danuglipron	Oral nonpeptide GLP-1 RA	Phase 2 completed	~11% WL; high discontinuation	Oral	[63]
Bimagrumab	Activin receptor II inhibitor + GLP-1	Phase 2-3	Improves body composition (fat loss with lean mass preservation)	Injectable (IV/SC, depending on trial)	[64]
GDF-15 analogues	Appetite regulation via stress response pathways	Early phase	Under investigation	Injectable (SC, investigational)	[65,66]

## Conclusions

Obesity is now recognized as a chronic, relapsing, multifactorial disease shaped by genetic, neuroendocrine, environmental, behavioral, and social determinants. Its pathogenesis involves complex interactions among biological systems, including the gut microbiome and early-life exposures. The consequences are systemic, impairing quality of life, increasing morbidity, and imposing high societal costs. Recent advances, such as GLP-1 and GIP agonists, endoscopic interventions, and AI-guided precision medicine, have transformed treatment paradigms. Emphasis is also shifting toward early prevention, microbiome-targeted strategies, and individualized care. Nonetheless, challenges persist. Weight stigma, limited access to care, and a singular focus on weight loss undermine outcomes and equity. In LMICs, care gaps are especially pronounced. Addressing the obesity epidemic requires a compassionate, interdisciplinary approach that moves beyond weight to holistic health. By integrating biology, behavior, and policy in a patient-centered framework, we can shift from reactive management to proactive, equitable, and enduring solutions.
